# Sustainable All-Cellulose Biocomposites from Renewable Biomass Resources Fabricated in a Water-Based Processing System by the Vacuum-Filtration-Assisted Impregnation Method

**DOI:** 10.3390/polym16131921

**Published:** 2024-07-05

**Authors:** Özkan Yapar, Petteri Piltonen, Ajra Hadela, Aleksandra Lobnik

**Affiliations:** 1 Faculty of Mechanical Engineering, University of Maribor, Smetanova Ulica 17, 2000 Maribor, Slovenia; aleksandra.lobnik@um.si; 2 Institute for Environmental Protection and Sensors (IOS) Ltd., Beloruska Ulica 7, 2000 Maribor, Slovenia; ajra.hadela@ios.si; 3 Fibre and Particle Engineering Research Unit, University of Oulu, P.O. Box 4300, 90014 Oulu, Finland; petteri.piltonen@gmail.com

**Keywords:** all-cellulose composites (ACCs), NaOH/urea/water solvent system, full dissolution of cellulose, dissolving pulp, regenerated cellulose

## Abstract

The increasing awareness of global ecological concerns and the rising sustainability consciousness associated with the manufacturing of non-renewable and non-biodegradable composite materials have led to extensive research on product and process developments of more sustainable, environmentally friendly, and fully biodegradable biocomposites for higher-value end-use applications. All-cellulose composites (ACCs) are an emerging class of biocomposites, which are produced utilizing solely cellulose as a raw material that is derived from various renewable biomass resources, such as trees and plants, and are assessed as fully biodegradable. In this study, sustainable ACCs were fabricated for the first time based on the full dissolution of commercially available sulfite dissolving (D) pulps as a matrix with concentrations of 1.5 wt.% and 2.0 wt.% in an aqueous NaOH–urea solvent, and they were then impregnated on/into the pre-fabricated birch (B), abaca (A), and northern softwood (N) fiber sheets as reinforcements by the vacuum-filtration-assisted impregnation approach. This research aimed to investigate the effects of the impregnated cellulose matrix concentrations and types of the utilized cellulose fiber reinforcements (B, A, N) on the morphological, crystalline, structural, and physio-mechanical properties of the ACCs. The highest degrees of improvements were achieved for tensile strength (+532%, i.e., from 9.24 MPa to 58.04 MPa) and strain at break of the B fiber-reinforced ACC B_1.5_ (+446%, i.e., from 1.36% to 4.62%) fabricated with vacuum impregnation of the 1.5 wt.% cellulose matrix. Noticeably, the greatest improvements were attained in strain at break of the A and N fiber-reinforced ACCs A_2.0_ (+218%, i.e., from 4.44 % to 14.11%) and N_2.0_ (+466%, i.e., 2.59% to 14.65%), respectively, produced with vacuum impregnation of the 2.0 wt.% cellulose matrix. The study highlights the diverse properties of the all-cellulose biocomposite materials that could, expectedly, lead to further development and research for upscaled production of the ACCs.

## 1. Introduction

Due to the increasing environmental concerns and awareness of the detrimental impacts of synthetic materials, there is a growing interest and demand for developing eco-friendly and cost-efficient biocomposites in many fields [1,2]. Cellulose is a polysaccharide and is the most abundant natural, renewable, biocompatible, and biodegradable biopolymer to be found in nature [3,4,5]. Primary sources of the cellulose are woods [6] such as softwoods, e.g., spruce [6], and hardwoods, e.g., birch [7], plants [8] or agricultural residues [9], tunicates [10], specific types of algae [11], and bacteria [12]. In this context, wood pulps remain the most important raw material source for the processing of cellulose, most of which are utilized extensively for the manufacturing of papers and paperboards [13].

All-cellulose composites (ACCs) are an emerging class of bio-based composites where both the matrix and reinforcement consist of non-derivatized cellulose [14]. ACCs have emerged as an interesting candidate in the field of biocomposites because their matrix and reinforcement phase are entirely compatible with each other owing to being chemically similar, or even identical, which permits an efficient stress transfer and robust adhesion at their interface [15,16]. ACCs can also exhibit optical transparency [1,17,18,19,20] and biodegradability [21]. This new class of biocomposites is recyclable, exhibiting low toxicity and low density, and they may offer sufficient mechanical, gas barrier, and optical properties [22]. These all-cellulose composites (ACCs) have been described as ‘interfaceless’ biocomposites, due to their perfect interfacial compatibility [23,24,25], a critical and valuable point for ACCs, because conventional biocomposites face challenges with their poor interfacial adhesion between a hydrophilic plant-based reinforcement and a hydrophobic matrix, often resulting in weakened mechanical performance and problems in recycling owing to their heterogeneous mixture [22,26].

Cellulose is not applicable for melt processing, but it can be dissolved with appropriate solvents [27]. ACCs can be prepared via two different ways: (a) by the partial dissolution method, where the cellulose fiber network is partially dissolved in a solvent followed by its regeneration, forming a matrix phase in situ around the remaining undissolved fiber network, with this route being used by the vast majority of studies [1,14,17,28,29,30,31,32,33,34], and (b) by the full dissolution method, where the cellulose is dissolved completely in a solvent system, subsequently impregnated and regenerated in the presence of undissolved cellulose reinforcement [27,35,36,37,38,39,40], for which relatively lower numbers of studies were published. The former procedure is also referred to as selective surface or (surface) selective dissolution [1,41,42], solvent or in situ welding [43,44], controlled dissolution [45], or the one-step method [46,47]. The latter pathway is also called impregnation [48], or the two-step method [49].

Several types of solvents have been reported for the fabrication of ACCs via the full dissolution method, including lithium chloride/*N,N*-dimethylacetamide (LiCl/DMAc) [35,37,38,50,51], sodium hydroxide (NaOH)/water [52], NaOH/urea/water [38,39,40,53,54,55,56], LiOH/urea/water [57], N-methyl-morpholine oxide (NMMO)/water [27,58], and ionic liquids [49,59,60,61]. Among them, the NaOH/urea aqueous solution is non-volatile, environmentally friendly, and inexpensive [62] (especially in comparison with imidazolium- and pyridinium-based ionic liquids) [14].

Only several papers have been published [38,39,40,53,54,55,56] on the production of ACCs via the full dissolution pathway using an aqueous NaOH/urea solvent system. The vast majority of these studies reported on ACCs that were fabricated in film forms where cotton linter pulp was used as a matrix reinforced with tunicates [38], cotton nanowhiskers [38,53], TEMPO-oxidized cellulose nanofibrils [39], and cut forms of wet-spun regenerated cellulose [56] and ramie fibers [54] using a simple casting method in which 5 wt.% sulfuric acid (H_2_SO_4_) was utilized as a coagulant (anti-solvent) [38,53,54,56]. Another published study reported on ACCs that were fabricated in gel forms, where cotton linter pulp was used as a matrix reinforced with cellulose nanowhiskers using the injection method in which running water was utilized as an anti-solvent [55]. Another study reported was for ACCs where the cotton linter pulp used as a matrix was reinforced with cotton woven fabrics with the use of the dip-padding method in which water was utilized as an anti-solvent [40].

The novelty of this present study was to explore the feasibility of utilizing various kinds of micron-sized cellulose fibers for the preparation of ACCs with an aqueous NaOH/urea system by the full dissolution method. Table 1 presents the ACCs using full dissolution in the NaOH/urea/water solvent system and their published mechanical properties with (dis)improvement rates. In this study, the strain at break values of the listed ACCs are described with percentages (%), while the alterations in their stress at break (MPa), elastic modulus (GPa), and strain at break (%) values, based on their reference sample, are represented with (dis)improvement rates, i.e., ±%.

Here, we report on different types of environmentally friendly all-cellulose composites (ACCs) which were prepared successfully based on the full dissolution of commercially available sulfite dissolving pulps in a NaOH/urea/water (7/12/81 wt.%) solution. The transparent dissolved cellulose matrices in concentrations of 1.5 wt.% and 2.0 wt.% were impregnated on/into pre-fabricated birch (B), abaca (A), and northern softwood (N) fiber reinforcement sheets, employing a similar vacuum filtration method, which has been already reported for use in cellulose nano-paper preparations [64,65]. The prepared ACCs were analyzed using field emission electron microscopy (FE-SEM), X-ray diffraction (XRD), and a universal tensile testing machine. The results show that the mechanical properties of the cellulose fiber reinforcement sheets (B, A, N) were significantly enhanced with remarkable improvement rates, resulting in various forms of the ACCs (B_1.5_, B_2.0_, A_1.5_, A_2.0_, N_1.5_, N_2.0_) (Table 1). The highest levels of enhancements were acquired for tensile strength (+532%) and elongation at break (+446%) of the birch (B) fiber reinforcements with vacuum impregnation of a 1.5 wt.% cellulose matrix (ACC B_1.5_). Noticeably, the highest levels of improvements were attained for the elongation at break values of the abaca (A) (+218%) and northern softwood (N) (+466%) fiber reinforcements with vacuum impregnation of the 2.0 wt.% cellulose matrix, i.e., ACC A_2.0_ and N_2.0_, respectively. The crystallinity index (*Crl*, *%*) values, calculated with the XRD measurements, confirmed the full dissolution of the sulfite dissolving pulp fibers by their analysis in the form of regenerated cellulose (RC) films (F_1.5_, F_2.0_), which were produced only for that characterization. The successful impregnations and formations of the ACCs were confirmed based on the alterations in the *Crl*, *%* values. The primary aim of this research was to explore the various properties of all-cellulose composites (ACCs) derived from different biomass resources, which were impregnated with two distinct fully dissolved cellulose matrix solutions of commercially available sulfite dissolving pulps, using the full dissolution method in an aqueous NaOH/urea solvent system. The main objectives of the presented research were the assessment of the effects of wood- and plant-leaf-based pulp fiber sheets, together with two different concentrations of the used fully dissolved cellulose matrix solutions, on the final properties of the ACCs, accordingly identifying the optimal processing conditions of the designed lab protocol. For the conduction of the respective tests and characterizations, a total number of 88 samples were used in this study.

The study highlights the different properties of the all-cellulose biocomposites from the birch-, northern softwood-, and abaca (leaf)-based cellulose fiber pulps, by the vacuum-filtration-assisted impregnation method presented here, paving the way for further development and research for upscaled production of the ACCs, targeting green packaging and environmental applications such as fully bio-based mulching and wrapping materials.

## 2. Materials and Methods

### 2.1. Materials

Birch (B), abaca (A), northern softwood (N), and sulfite dissolving (D) pulps were used as reinforcements (B, A, N) and matrices (D) for fabrications of the all-cellulose composites (ACCs), which were provided by the National Institute of Standards and Technology (NIST, Gaithersburg, MD, USA), Glatfelter (Gernsbach, Germany), and Domsjö Fabriker AB (Örnsköldsvik, Sweden), respectively. The degrees of polymerization (DPs) of the produced cellulose fiber sheets (B, A, N) and the sulfite dissolving (D) pulps were 1026, 1279, 1179, and 580.73, respectively, which were calculated from their limiting viscosity numbers [η] according to the standard methods ISO 5351:2004 (E) [59]. and ASTM D1795-13 [66]. The average fiber lengths of the B, A, and N pulps were 0.9 mm, 2.3 mm, and 3.5 mm, respectively, measured according to TAPPI/ANSI T 271 om-12 [61].

Sodium hydroxide (NaOH, reagent grade, ≥98%, pellets, anhydrous), urea (ReagentPlus^®^, ≥99.5%, pellets), and distilled water were used to prepare the solvent mixture to dissolve cellulose fibers of the sulfite dissolving (D) pulps. A cupri-ethylenediamine (CED) solution (1.0 M in H_2_O) was utilized in the measurement of limiting viscosity numbers [η], to determine the DP values of the utilized sulfite dissolving (D) pulps and the fabricated cellulose fiber sheets (B, A, N). All the utilized chemicals were purchased from Sigma Aldrich (Karlsruhe, Germany) and used as received without further purification.

### 2.2. Cellulose Reinforcement Preparation

The cellulose fiber sheets (average grammage of 80 g/m^2^) were prepared according to ISO 5269-1:2005 [62]. from birch (B), abaca (A), and northern softwood (N) pulps by the conventional sheet forming method and coded accordingly as B, A, and N, respectively. The sheets were pressed at 0.4 MPa with a sheet pressing machine (AB Lorentzen & Wettre, Kista, Sweden), dried inside a drying chamber (Oy Lorentzen & Wettre AB, Finland AB, Helsinki, Finland) at 65 °C for 24 h to remove residual moisture, and were stored in a desiccator until further use.

### 2.3. Cellulose Matrix Preparation

The solvent mixture for full dissolution of the sulfite dissolving (D) pulp fibers was prepared by dissolving the NaOH and urea in distilled water (7:12:81 wt.%) by using a magnetic stirrer in a glass beaker at room temperature for 20 min.

At first, the sulfite dissolving (D) pulps were dried in an oven at 105 °C for 15 h to remove residual moisture, and they were subsequently stored in a desiccator for 2 h. Thereafter, they were shredded into small pieces manually to ease the dissolution process. Dissolution of the sulfite dissolving (D) pulp pieces was carried out in a glass beaker submerged inside a cooling bath (Lauda Eco Silver, RE 1050, LAUDA Dr. r. Wobser GmbH & Co.KG, Lauda-Königshofen, Germany) at −12.6 °C. A high-shear mixer (UltraTurrax, IKA T25, IKA-Werke GmbH & Co. KG, Staufen, Germany) was placed into the beaker in the cooling bath to mix and disintegrate larger pieces of cellulose (D) at 3000–14,000 rpm for 45 min. The concentrations of the prepared dissolved cellulose solutions as a matrix were 1.5 wt.% and 2.0 wt.%. Subsequently, the completely dissolved cellulose portions were centrifuged (Beckman Coulter, Inc., Avanti J-26-XPI, Brea, CA USA) at 6000 rpm for 10 min at 5–10 °C in order to carry out degassing and to ensure full dissolution of the sulfite dissolving (D) pulp fibers. Eventually, these centrifuged cellulose matrices (5–10 °C) were placed again in the cooling bath at −12.6 °C for 1 h to make them ready for fabrication of the ACCs.

### 2.4. Preparation of the ACCs

**Figure 1:**
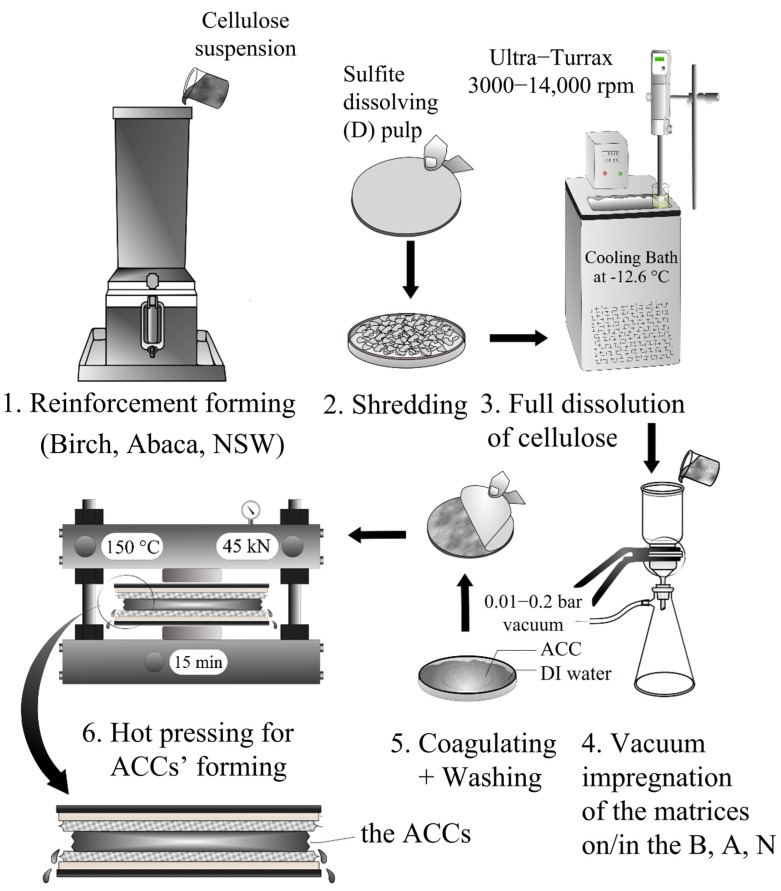
Schematic illustration of ACCs’ preparation by the vacuum-filtration-assisted impregnation method.

The cellulose fiber reinforcement sheets prepared from birch (B), abaca (A), and northern softwood (N) pulps were impregnated using a vacuum filtering of fully dissolved matrices of the sulfite dissolving (D) pulps with 1.5 wt.% and 2.0 wt.% concentrations (B_1.5_, A_1.5_, N_1.5_ and B_2.0_, A_2.0_, N_2.0_). The schematic illustration of the ACCs’ preparation is presented in Figure 1. To form the ACCs, each fiber reinforcement sheet (B, A, N) was inserted onto a metal filter part and poured over the dissolved cellulose (D) matrices at a 0.01–0.2 mbar vacuum pressure at room temperature for 30–60 s. The impregnated cellulose fiber reinforcement sheets were inserted into a water bath together with the removable metal filter part to avoid their breakage. The impregnated cellulose samples were coagulated and washed by immersing them in a bath of distilled water at room temperature for 72 h until the pH of the washing solution was neutral, resulting in the regeneration of these samples. After washing, the regenerated cellulose samples were placed between blotting papers, Mylar^®^ films, and aluminum plates and dried with a hot press (Fontijne Press, LabEcon 300, Fontijne Presses BV, Vlaardingen, The Netherlands) at 150 °C under 45 kN pressure for 15 min. Eventually, the ACC samples (B_1.5_, A_1.5_, N_1.5_ and B_2.0_, A_2.0_, N_2.0_) were produced, and they were stored for 48 h at 23 °C and 50% ± 2 relative humidity (RH) prior to their structural and mechanical characterizations.

### 2.5. Regenerated Cellulose Film Preparation

Only for the XRD crystallinity measurements of the fully dissolved cellulose matrix solutions (1.5 wt.% and 2.0 wt.%) of the sulfite dissolving (D) pulps, regenerated cellulose films (F_1.5_, F_2.0_) were fabricated by casting of the cellulose matrices into Petri dishes to form their hydrogels, which were air dried at room temperature for 48 h. The air-dried films were coagulated and washed in a distilled water bath at an ambient temperature for 24 h. The water was changed circa 5 times until its pH was neutral and all the chemicals and residues were removed. The regenerated cellulose (RC) films (F_1.5_, F_2.0_) were inserted into clean paper sheets, meshes, and metal plates for 72 h at room temperature with 50% ± 2 RH for an additional drying process.

## 3. Characterizations

### 3.1. Degree of Polymerization (DP)

The limiting viscosity numbers (η) of the sulfite dissolving (D) pulps and of all the cellulose fiber reinforcement sheets (B, A, N) were analyzed by immersing them in a cupri-ethylenediamine (CED, or cuen) solution, according to the standard methods ISO 5351:2004 (E) and ASTM D1795-13 [66]. The flow rate was measured with a capillary viscometer at 25 °C. Mean values were calculated from five samples for each of the cellulose samples.

The degree of polymerization (DP) was calculated through its correlation with the limiting viscosity of the pulps according to Equation (1) [67]:

(1) $${DP}^{0.905}=0.75\times [\eta ]$$

### 3.2. Thickness and Density

The thicknesses (µm) of the birch (B), abaca (A), and northern softwood (N) fiber reinforcement sheets and the ACC samples (B_1.5_, A_1.5_, N_1.5_, B_2.0_, A_2.0_, N_2.0_) assessed in this study were measured using a digital caliper (Lorentzen and Wettre, AB Lorentzen and Wettre, Kista, Sweden) according to ASTM D645/D645M-97 [68] to carry out their tensile tests and to obtain data for their density calculations (g/cm^3^). All the measurements were conducted at room temperature at 50 ± 2 RH after their conditioning for at least 48 h.

For the density calculations, the fiber reinforcement sheets (B, A, N) and the produced ACCs (B_1.5_, A_1.5_, N_1.5_ and B_2.0_, A_2.0_, N_2.0_) were cut into circular shapes (cm^2^), their thicknesses (µm) were measured from 25 locations, and mean values were used to calculate the density of each one (g/cm^3^).

### 3.3. Field Emission Scanning Electron Microscopy (FE-SEM)

The surfaces and cross-sections of all the used fiber reinforcement sheets and the ACC samples were imaged using a field emission scanning electron microscope (Zeiss Sigma and Zeiss Ultra Plus; Carl Zeiss Microscopy GmbH, Jena, Germany) equipped with a secondary electron detector at an accelerating voltage of 5 kV. Pt/Pd was deposited on the surface of all these samples to prevent the charging of the specimen. For the cross-sectional imaging, each sample was cut with a razor blade to reveal the complex structure.

### 3.4. X-ray Diffraction (XRD)

The crystalline structures of the cellulose fiber reinforcement sheets (B, A, N), RC films (F_1.5_, F_2.0_) and the ACCs (B_1.5_, A_1.5_, N_1.5_ and B_2.0_, A_2.0_, N_2.0_) were analyzed using wide-angle X-ray diffraction (WAXD) at an ambient temperature. Each of the samples was mounted on a solid circular holder, and a proportional counter detector was set to collect the data. X-ray patterns for all the samples were acquired with a Siemens D5000 diffractometer using monochromator-filtered Cu-Kα radiation (λ = 0.1542 nm) and scanning in the region of 2θ from 5 to 35° at a scanning speed of 0.85° min^−1^. The accelerating voltage and current were set at 40 kV and 40 mA, respectively.

The crystallinity index (*Crl,* %) values of all the samples were estimated by the method of Segal and co-workers [69] using the following relationship based on presented intensity data:

(2) $$Crl\;(\%)=\left(I_{200}-I_{am}\right)/I_{200}\times 100$$

where I_200_ is the peak intensity corresponding to both the amorphous and crystalline fractions of cellulose I_200_, and I_am_ is the peak intensity of the amorphous fractions.

### 3.5. Mechanical Testing

The mechanical properties of the cellulose fiber reinforcement sheets (B, A, N) and the ACCs (B_1.5_, A_1.5_, N_1.5_ and B_2.0_, A_2.0_, N_2.0_) were examined using a universal testing machine (Instron 5544, Norwood, MA, USA) equipped with a 100 N load cell according to ASTM D882 [70]. All the specimens were cut by a fine blade as rectangular strips with 5 mm width. Prior to testing, all the samples were conditioned at 50 ± 2 relative humidity (RH) and 23 °C for a minimum of 48 h according to ASTM E104 [71]. The thicknesses of these samples were measured using a digital caliper (Lorentzen and Wettre, AB Lorentzen and Wettre, Kista, Sweden). Five different locations from each strip were measured, and mean values were used in the calculations. All the specimens were tested at a constant cross-head speed of 4 mm/min and a grip distance of 30 mm. A minimum of five replicates were tested for each sample.

## 4. Results and Discussion

### 4.1. Degree of Polymerization (DP)

The degrees of polymerization (DPs) of the birch (B), abaca (A), and northern softwood (N) fiber sheets used for the fabrication of the all-cellulose composite (ACC) samples in this study were 1026, 1279, and 1179, respectively. The obtained DP value for the sulfite dissolving pulp (D), which was used for full dissolution in the aqueous NaOH/urea solvent system, was 580.73. A similar result was published with a similar DP of used cellulose by Chen et al., who achieved the full dissolution of cotton linter pulps (620 of DP) using a NaOH/urea/water solvent [72]. The DP (620) of their cellulose source was even higher than that of the sulfite dissolving pulp (581) in our study, which indicates the suitability of our cellulose for complete dissolution.

### 4.2. FE-SEM

**Figure 2:**
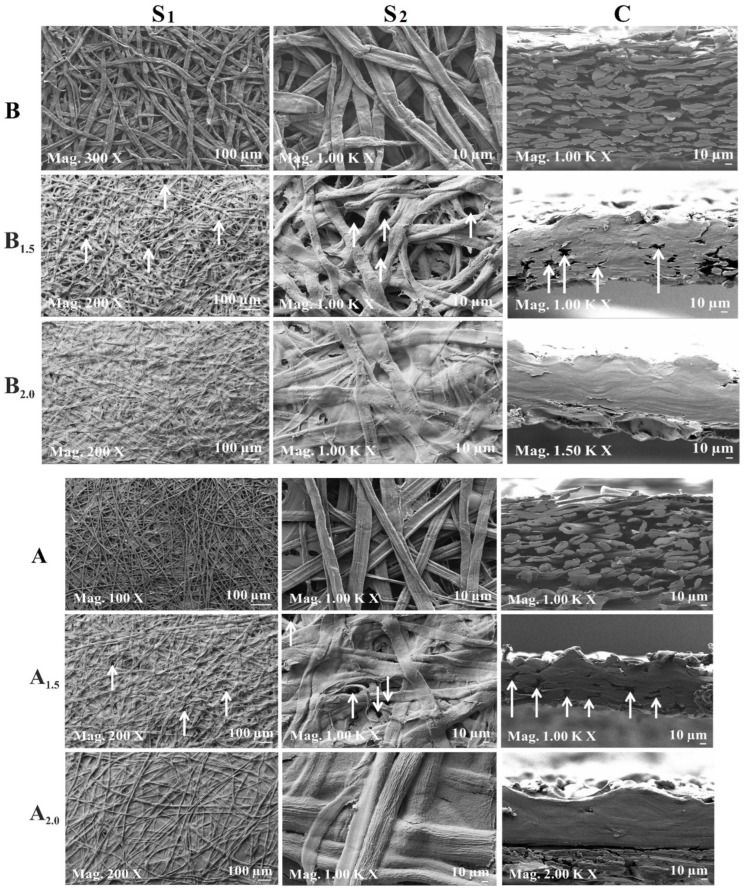

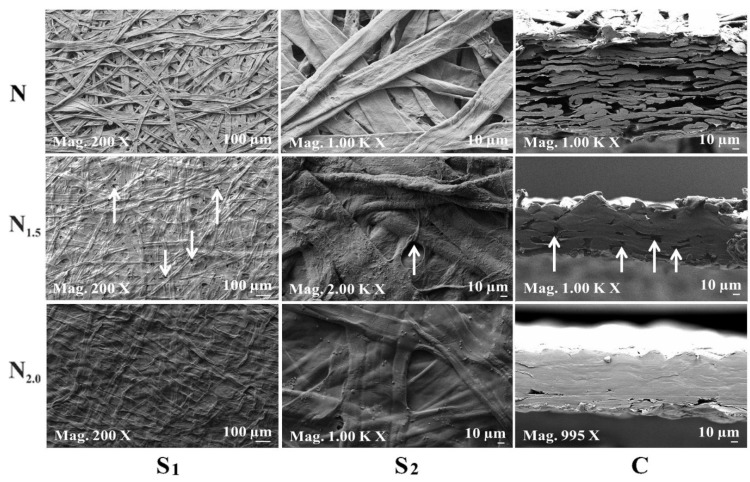
FE-SEM images of the all-cellulose composite samples (**B_1.5_**, **A_1.5_**, **N_1.5_**, **B_2.0_**, **A_2.0_**, **N_2.0_**) prepared from birch (**B**), abaca (**A**), and northern softwood (**N**) fiber reinforcement sheets impregnated with 1.5 wt.% and 2.0 wt.% of dissolved cellulose matrices using a vacuum-filtration set-up. (**S_1_**,**S_2_**) correspond to the surface, while C corresponds to the cross-sections of all the samples with 100 µm (**S_1_**) and 10 µm (**S_2_**,**C**) scale bars. The white arrows indicate some of the inter- or intra-laminar voids.

FE-SEM images of the produced cellulose fiber sheets (B, A, N) and the ACC samples (B_1.5_, A_1.5_, N_1.5_ and B_2.0_, A_2.0_, N_2.0_) are presented in Figure 2. Herein, the acquired micrographs in the first column on the left indicate a general overview of the surface morphology of all the samples (Figure 2S_1_)_,_ while the ones in the middle column represent a more detailed look on their surfaces with higher magnification (Figure 2S_2_). The obtained FE-SEM images in the right column present the cross-sectional structures of all the samples (Figure 2C). The fibrous structures of the birch (B), abaca (A), and northern softwood (N) fiber sheets can be seen clearly in both their surface (Figure 2S_1_,S_2_) and cross-sectional parts (Figure 2C) prior to the application of the vacuum-filtration-aided impregnation process with the dissolved cellulose matrix solutions. The reinforcing cellulose fibers (B, A, N) remained slightly visible on the surface of all the ACC samples (B_1.5_, A_1.5_, N_1.5_ and B_2.0_, A_2.0_, N_2.0_), indicating a successful impregnation without causing any degradation to the reinforcement fiber sheets (B, A, N) (Figure 2S_1_,S_2_). As a result of the impregnated 2.0 wt.% dissolved cellulose matrix solution on/into the cellulose fiber sheets (B, A, N), more uniform and compact filling of the micro-voids between fibers was seen in comparison to the impregnating effect of the 1.5 wt. % solution. Similar results were also observed by Kidwai et al. for the compactness of their ACC samples as a result of the impregnation of dissolved cellulose solutions (prepared by full dissolution with a NaOH/urea solvent system) in their SEM micrographs [40]. That result in our study could be elucidated due to the higher liquidity of the aqueous solution mixture of the 1.5 wt.% portions during the vacuum impregnation process in comparison with the 2.0 wt.% solution. Thus, that results in a lower amount of dissolved cellulose remaining on/in the impregnated cellulose fiber sheets (B, A, N) at the end of impregnation of the 1.5 wt.% matrix solution. Thereby, (more) voids are noticed on the cross-sectional micrographs (Figure 2C) of these all-cellulose bio-composite samples B_1.5_, A_1.5_, and N_1.5_ in comparison with samples B_2.0_, A_2.0_, and N_2.0_.

Figure 2C shows the cross-sections of all the ACCs prepared with 1.5 wt. and 2.0 wt.% dissolved cellulose matrices. Some inter-laminar voids were noticed on the cross-sections of these ACC samples (B_1.5_, A_1.5_, N_1.5_) and are pointed out with white arrows (Figure 2C). The ACCs composed of the impregnated 2.0 wt.% of dissolved cellulose (B_2.0_, A_2.0_, N_2.0_) facilitated a more compact and tightly intertwined structure with almost no delamination or cracks, indicating good compatibility between the fibers and the matrix (Figure 2C).

### 4.3. XRD

**Figure 3:**
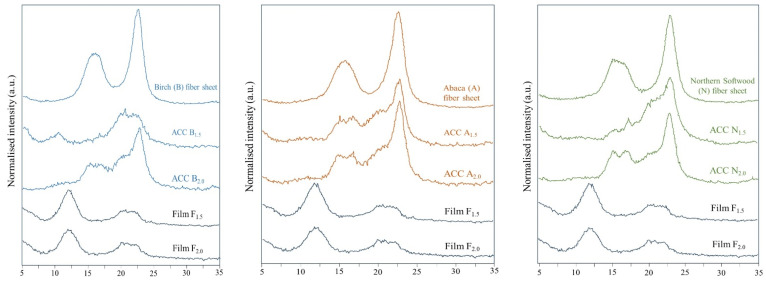
X-ray diffraction profiles of the ACCs (B_1.5_, A_1.5_, N_1.5_ and B_2.0_, A_2.0_, N_2.0_), indicating the alterations in the crystallinity compared with birch (B), abaca (A), and northern softwood (N) fiber sheets and prepared films (F_1.5_, F_2.0_) from the fully dissolved 1.5 and 2.0 wt.% cellulose matrix solutions.

Figure 3 depicts the X-ray diffraction patterns of the cellulose fiber reinforcement sheets (B, A, N), prepared all-cellulose composites (B_1.5_, A_1.5_, N_1.5_ and B_2.0_, A_2.0_, N_2.0_), and regenerated cellulose films (F_1.5_, F_2.0_) from the fully dissolved cellulose matrix solutions. The birch (B), abaca (A), and northern softwood (N) fiber sheets comprise typical natural cellulose with a crystal structure of cellulose I. In this study, the cellulose reinforcements (B, A, N) exhibited three peaks at 2θ = 14.8°, 16.4°, and 22.7° for (1$\overset{-}{1}$0), (110), and (200) planes that are typically characteristic of a cellulose I crystalline structure. Similar results were reported by Li et al. [73] for their use of different cellulose materials, which were dissolved in the aqueous NaOH/urea solvent system by the coagulation of water. The diffraction peaks of the regenerated cellulose films (F_1.5_, F_2.0_) at 2θ = 12.3°, 20.2°, and 22.3° were assigned to the (1$\overset{-}{1}$0), (110), and (200) planes of cellulose II crystalline structure, attributed to the typical regeneration of cellulose, as reported earlier. Similar kinds of regenerated cellulose films were reported by Li et al. [74] using cotton linter pulp, which was dissolved with an aqueous NaOH/urea solvent system and coagulated with water. The results indicate clearly that the cellulose reference films (F_1.5_, F_2.0_) prepared from sulfite dissolving (D) pulps were transformed successfully into cellulose II allomorphs. As represented in Table 2, the overall crystallinity index values of the F_1.5_ and F_2.0_ were found to be similar to each other. Similar results to these *Crl (%)* values (43.7–43.9%) in our study were reported by Qi et al. for their values (40–44%) of all-cellulose composite films prepared with the full dissolution method reinforced with cellulose nanocrystals [53].

Moreover, the X-ray diffraction profiles of the ACC samples indicate the presence of both cellulose I and II crystalline structures. Similar results were also observed in the reported study of Kidwai et al. for their cotton woven fabric-reinforced ACCs impregnated with fully dissolved cellulose solutions in a NaOH/urea solvent system. The typical reflection peaks at 2θ angles of 16° and 22° were noticed for all ACCs in their and our studies, as these patterns correspond to cellulose I, which comprises two distinctive crystalline forms, i.e., cellulose Iα (triclinic) and cellulose Iβ (monoclinic) [40]. In Figure 3, the distinctive peaks at 22 for all the ACC samples represent the (200) plane lattice, corresponding to both the crystalline and amorphous parts of the cellulose l, while the diffraction at 18.76° corresponds only to the amorphous part [40]. Interestingly, Kidwai et al. reported their ACC samples (dip-padding with 1.5% dissolved cellulose matrix) having a higher *Crl* (%) value (63.9%) in comparison with untreated cotton woven fabric reinforcement (65%). That phenomenon (the increase in the crystallinity index from 63.9% to 65%) indicates a different behavior than the ACCs presented in this study (decrease in the index compared with our B, A, and N reinforcements). The differences in these results could be due to a lack of hot pressing in the study of Kidwai et al. that might have resulted in different interactions between the dissolved cellulose and reinforcements. Another parameter could be the low amount of dissolved cellulose usage in their studies, as reported in their Results part [40]. Hot pressing is a crucial step in forming ACCs, and it results in smoother surfaces and fewer cavities compared to an air-dried regenerated cellulose structure, as was concluded clearly for the wood cellulose films fabricated with a NaOH/urea solvent system by Huan et al. They reported the contribution to the dramatically improved mechanical strength with the aid of a hot press for their RC films (which are in/directly connected with the crystallinity index values too) [75]. The crystallinity index (Crl %) of cellulose fiber reinforcement sheets (B, A, N) decreased from 73.1 for B to 44.4 for B_1.5_, from 76.8 for A to 60.6 for A_1.5_, and from 78.3 for N to 59.2 for N_1.5_ (Table 2).

Interestingly, the crystallinity index values for B_2.0,_ A_2.0,_ and N_2.0_ were 58.33, 61.48, and 66.19, respectively, which were higher than those for B_1.5,_ A_1.5,_ and N_1.5_. Theoretically, the crystallinity index values for B_2.0,_ A_2.0,_ and N_2.0_ were expected to be lower than those for B_1.5,_ A_1.5_, and N_1.5,_ due to the higher amount of regenerated cellulose. The results could be elucidated by the higher dissolving effect of the 1.5 wt.% cellulose solution, which might have dissolved the cellulose fiber sheets (B, A, N) during the impregnation phase, leading to partial dissolution of the fibers in the sheets, which could also be noticed from the disappearing of the typical peaks at (200) planes in the XRD pattern of B_1.5_. Similar results were reported by Quajai and Shanks (2009) when hemp fibers were used, especially with low crystallinity [27].

### 4.4. Mechanical Properties

**Figure 4:**
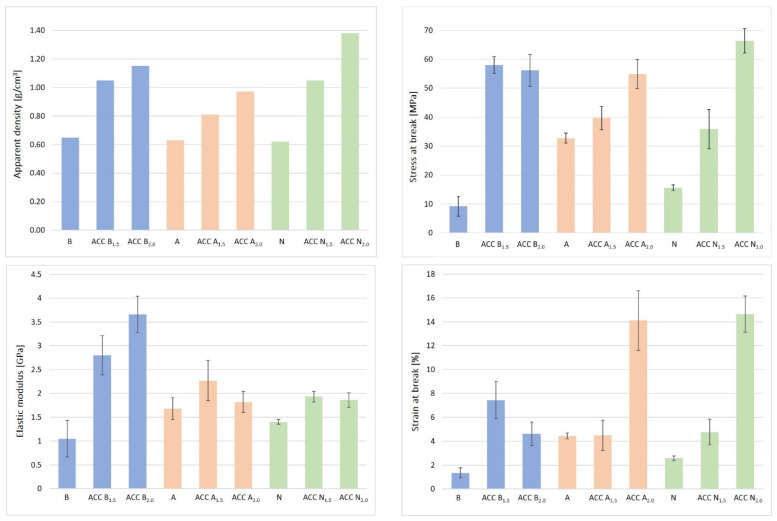
Apparent density (g/cm^3^), stress at break (MPa), elastic modulus (GPa), and strain at break (%) for all the samples.

The densities (g/cm^3^) and crystallinity index (*Crl* %) values, as well as the results from the mechanical tests of the birch (B), abaca (A), and northern softwood (N) fiber sheets and fabricated all-cellulose composite (ACC) samples (B_1.5_, A_1.5_, N_1.5_ and B_2.0_, A_2.0_, N_2.0_), are presented in Table 3. Figure 4 presents their stress–strain curves. It was observed that the densities of the utilized cellulose fiber sheets (0.65 g/cm^3^ for B, 0.63 g/cm^3^ for A and 0.62 g/cm^3^ for N) increased when the concentration of dissolved cellulose matrix solutions increased (from 1.5 wt.% to 2.0 wt.%) for forming the respective ACC samples, i.e., 1.05 g/cm^3^ for B_1.5_, 0.81 g/cm^3^ for A_1.5_, and 1.05 g/cm^3^ for N_1.5_ and 1.15 g/cm^3^ for B_2.0_, 0.97 g/cm^3^ for A_2.0_, and 1.38 g/cm^3^ for N_2.0_. Similarly, it was reported that the density of the biocomposite was increased by the addition of HA from 5% to 20%, as in the case of this presented study, i.e., the addition of the cellulose matrix concentrations from 1.5 wt.% to 2.0 wt.% for denser ACC samples [76]. For the samples of abaca (A) and northern softwood (N) fiber sheets, the higher concentration of the impregnated dissolved cellulose solutions resulted in higher tensile strength and higher strain at break values of the ACCs of A_2.0_ (54.9 MPa and 14.1%) and N_2.0_ (66.4 MPa and 14.6%) in comparison with the ACCs of A_1.5_ (39.7 MPa and 4.5%) and N_1.5_ (35.8 MPa and 4.8%). Interestingly, for the birch (B) fiber sheets, higher tensile strength (MPa) and higher strain at break (%) were acquired for the ACC sample of B_1.5_ (58.0 MPa and 7.4%) in comparison with the ACC sample of B_2.0_ (56.2 MPa and 4.6%). The birch (B) fiber sheets had a lower tensile strength (9.2 MPa) and strain at break value (1.3%) compared to abaca (32.7 MPa and 4.4%) and northern softwood fiber sheets (15.6 MPa and 2.6%).

Noticeably, the strain at break (%) values of the northern softwood (N) fiber sheet increased from 2.59 to 14.65 with a +466% improvement rate for the ACC of N_2.0._ That is a remarkable achievement and could be important, especially if the ACCs are utilized in a new class of packaging and biomaterials. The tensile strength of the N sheet was enhanced from 15.64 MPa to 66.43 MPa with the use of the 2.0 wt.% dissolved cellulose matrix, resulting in the ACC sample of N_2.0,_ which was the highest value (66.43 MPa) attained in this study. The tensile strength and strain at break of the A fiber sheet (32.7 MPa and 4.4%) improved greatly with impregnation with the 2.0 wt.% dissolved solution, resulting in the ACC sample of A_2.0_ (54.9 MPa and 14.1%) with also noticeably remarkable rates of enhancements, i.e., +68% and +218%, respectively.

A different behavior was observed for the birch (B) fiber sheets. The tensile strength and strain at break of the ACC B_1.5_ samples yielded higher values than the ACC B_2.0_ samples, i.e., from 9.24 to 58.04 and 1.36% to 7.43%, respectively_._ This was the highest level of improvement in this study, with the sample of B_1.5_ with around +532% and +446% in comparison with the tensile strength and strain at break values of the abaca (A) (54.0 MPa and 14.1%) and northern softwood (N) (66.4 MPa and 14.6%) fiber sheets which were impregnated with 2.0 wt.% dissolved cellulose. That interesting result for the B_1.5_ samples could be explained by means of the relatively lower DP of the birch fiber sheets in this study, which might have resulted in a partial dissolution of the reinforcement fibers (B) in the 1.5 wt.% cellulose matrix solution at −12 °C temperature during the vacuum impregnation process. Since the surface selective dissolution of the cellulose with the NaOH/urea/water solvent could be associated intrinsically with a sort of mercerization process, the accessed parts of these birch (B) fibers of the B_1.5_ specimens could have been transformed to cellulose II after coagulating and washing with water. Thus, their dissolved cellulose part complexes (B_1.5_) might have formed hydrogen bonds with the etched parts of the birch fibers. That phenomenon could have occurred as a consequence of the intercalation of sodium ions within the crystal structure and the irreversible forming of sodium–cellulose complexes. Therefore, the synergistically reinforcing effect might have arisen from the interfacial adhesion between the dissolved and undissolved assemblies of the birch fiber sheets. As a result, that might have permitted more stress transfer capabilities in the impregnated B_1.5_ sample, thus imparting an effective reinforcement effect to the sample as a result of the applied hot press for the processing of the wet fibers.

**Figure 5:**
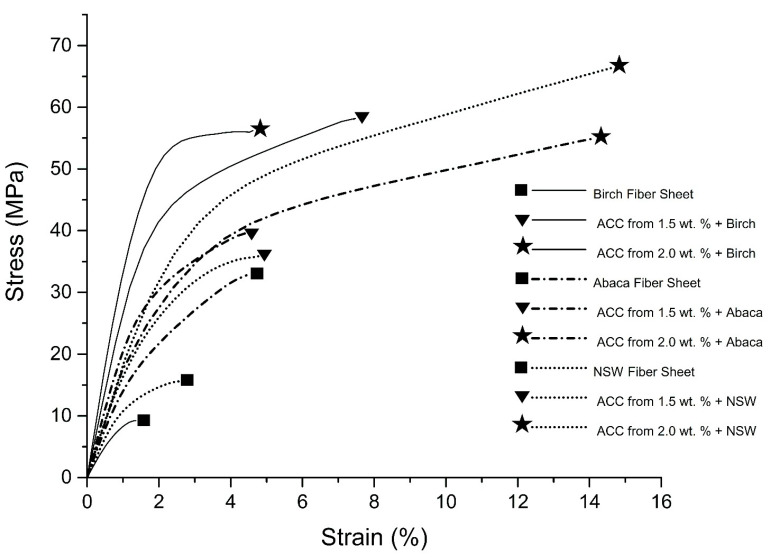
Stress–strain curves of the birch (B), abaca (A), and northern softwood (N) fiber reinforcement sheets and all-cellulose bio-composite samples produced with impregnation of 1.5 wt. % (B_1.5_, A_1.5_, N_1.5_) and 2.0 wt. % (B_2.0_, A_2.0_, N_2.0_) dissolved cellulose matrices.

In summary, a significant level of improvements was noticed for all the reported tensile properties of all the ACC samples fabricated in this study, as shown in Table 1 and Figure 5. On that point, the ACC preparation protocol of the study by Kidwai et al. is the closest to our study, in which a slight decrease was reported for the Young’s modulus values of their samples [40]. That kind of result could be attributed to the lack of hot pressing in their study and lower concentration of dissolved cellulose solution impregnated (i.e., 0.5 and 1.5%) in comparison with our study (i.e., hot pressing at 150 °C under 45 kN pressure for 15 min). Huan et al. reported the dramatically improved mechanical strength of their regenerated cellulose films prepared by the NaOH/urea solvent method with the aid of the hot press compared with air-drying in the conclusions of their study [75]. Furthermore, Uusi-Tarkka et al. published an interesting study on how variations in the hot press temperatures (70 °C to 150 °C) affect the mechanical properties of their ACC laminates manufactured by a NaOH/urea solvent system. The results for their ACC samples indicated that a higher temperature during the hot press process increased the tensile strength. The elongation of their all-cellulose composites decreased when higher hot press temperatures were applied [77]. That means not only applying the hot press but also optimizing its parameters plays a significant role in affecting the final mechanical properties of the ACC samples. Although our study applied hot pressing to form the ACC samples, in our future studies, different hot press conditions will be examined to research the thermal–mechanical and physio-mechanical properties of ACCs as a first study of its kind, as described further in the Future Perspectives of this Study.

The cellulose matrix solution with a concentration of 2 wt.% was more saturated (for the ACC samples of B_2.0_) and, presumably, had a lower capability to dissolve cellulose in comparison with the 1.5 wt.% solution when they were impregnated on/into the birch (B) fiber sheets for the samples of B_1.5_. Thus, it would have resulted in higher tensile properties for the produced ACC samples of B_1.5_ than for those of B_2.0_. In comparison with the abaca (A) and northern softwood (N) fiber sheets which were impregnated with 2.0 wt.% dissolved cellulose portions (ACCs of A_2.0_ and N_2.0_), the elastic modulus of the B_2.0_ samples showed the highest value with the highest improvement from 1.05 to 3.66 GPa. The improvement of stress at break, elastic modulus, and strain at break values all together is challenging to achieve for biocomposites, but for the N_2.0_ samples, all the tensile properties of the northern softwood (N) fiber sheets were enhanced in this study. As a result, the 2 wt. % cellulose matrix solutions facilitated higher tensile properties of the A_2.0_ (54.9 MPa) and N_2.0_ (66.4 MPa) samples in comparison with A_1.5_ (39.6 MPa) and N_1.5_ (35.8 MPa) samples impregnated with 1.5% of the solution matrix. This result could be attributed to the formation of the fewest voids for the ACC samples of A_2.0_ and N_2.0_ compared with the ACCs of A_1.5_ and N_1.5_.

## 5. Conclusions

A kind of green all-cellulose bio-composites composed entirely of sustainable cellulose resources with highly improved mechanical properties has been fabricated successfully via the vacuum-filtration-assisted impregnation method with a low environmental impact, a cost-efficient process (especially in comparison with ionic liquids), and an easy-to-handle aqueous NaOH/urea solvent. From the performed analyses. we concluded the following:

(i) Innovative Fabrication Method: The vacuum-filtration-assisted impregnation pathway for impregnating different kinds of cellulose fiber sheets from the pulps with completely dissolved cellulose solution media in hydrogel forms obtained by the NaOH/urea/water solvent system as a promising approach for ACC preparation is reported here for the first time.

(ii) Eco-Friendly and Cost-Efficient Process: The presented fabrication method allows for simple, eco-friendly, and cost-effective regeneration (coagulation) of the prepared ACCs only by means of water baths. Thus, a further contribution to the economic and environmental sustainability of the manufacturing protocol would be expected strongly.

(iii) Improved Material Compatibility and Integration: The impregnation, integration, and interfacial compatibility of the acquired ACCs were exhibited throughout the cellulose sheets (B, A, and N) due to the structural similarities of cellulose I and cellulose II with strong hydrogen bonds between them.

(iv) Enhanced Mechanical Properties: This facile method improved the mechanical performance of all the used cellulose sheets markedly. The ACCs prepared in this study showed the highest improvement values for all the tensile tests reported in this solvent system and fabrication method until now, including the following results:

○ A 532% increase in stress at break for sample ACC B_1.5_ (birch fiber sheet reinforced with a 1.5 wt.% dissolved cellulose matrix) from the initial 9.24 MPa to 58.4 MPa;
○ A 248% increase in elastic modulus for sample ACC B_2.0_ (birch fiber sheet reinforced with a 2.0 wt.% dissolved cellulose matrix) from the initial 1.05 GPa to 3.66 GPa;
○ A 466% increase in strain at break for sample ACC N_2.0_ (northern softwood
○ fiber sheet reinforced with a 2.0 dissolved cellulose matrix) from the initial 2.59% to 14.65%.

(v) Potential for Upscaling: The demonstrated protocol exhibited high potential as a viable approach for upscaling the production of fully bio-based, reasonably bendable, possibly fully recyclable and biodegradable, yet noticeably strong ACCs (upon maintaining the meta-stable character and behaviors of full cellulose matrix solutions). This summarized Section 5 encapsulates the core achievements and implications of our research effectively, setting the stage for future developments in the field of sustainable material science.

### Future Perspectives of this Study

Exploring the effects of hot press conditions such as time, temperature, and pressure on the final mechanical properties of the respective ACC samples could be an avenue for experimental investigation. Future studies could also explore the novelty of incorporating nano-fibrillated cellulose (NFC) and/or nanocrystalline cellulose (NCC) sequentially into fully dissolved cellulose matrix solutions, to assess their impact on the physical and thermal–mechanical properties of the final all-cellulose (nano)composites. Additionally, examining the thermal stability of the projected samples using thermogravimetric analyses (TGA) could provide valuable insights into their performance under various thermal conditions.

As the first of its kind, the ACC manufacturing approach detailed in this study could be applied, with or without vacuum assistance, to other two-dimensional cellulosic fiber assemblies, such as recycled paper sheets from pulp–paper industries, non-wovens, and woven fabrics (for example, end-of-life cotton textile waste from denim fractions). This adaptation could facilitate the production and development of novel eco-friendly ACC laminates. Implementing this technique could represent a significant step toward a circular resource economy by enabling the upcycling of waste materials into valuable new products. This approach not only enhances sustainability, but also aligns with global efforts to reduce the environmental impact through innovative material reuse and recycling strategies.

Additionally, several pioneering studies could be carried out for our ACCs in the scope of our experimental protocol, such as a life cycle cost analysis (LCCA) and fracture mechanics and molecular dynamics simulations. For instance, the noticed crack paths in the ACC samples could be analyzed for their crack initiation and growth with electron backscatter diffraction mapping. The method of thermal desorption spectroscopy could be utilized for in-depth investigation of our ACCs, too. Notably, to the best of our knowledge, simulation studies have not yet been reported for all-cellulose composites using the solvent system and protocol presented in our study; thus, this could be another novel study.

## Figures and Tables

**Table 1:** Mean values of mechanical properties reported for the all-cellulose composites (ACCs) using the full dissolution method in a NaOH/urea/water solvent system in comparison to the mechanical properties of the ACCs presented in this study.

Matrices of the ACCs	Reinforcements of the ACCs	Immobilization Method (and Anti-Solvent)	Stress at Break (MPa) (±%)	Elastic Modulus (Gpa) (±%)	Strain at Break (%) (±%)	Ref.
Cotton linter pulp (DP: 500)	Regenerated cellulose (RC) film (reference without reinforcement)	Casting method (5 wt.% H_2_SO_4_)	87.0	3.92	9.5	[53]
	Cellulose nanowhiskers (CNWs: 10 wt.%) reinforced ACC films	Casting method (5 wt.% H_2_SO_4_)	124.0 **+47%**	5.10 **+30%**	6.0 **−37%**	
	Cellulose nanowhiskers (CNWs: 20 wt.%) reinforced ACC films	Casting method (5 wt.% H_2_SO_4_)	117.0 **+34%**	5.87 **+50%**	4.0 **−58%**	
Cotton linter pulps (DP: unspecified)	RC film (reference without reinforcement)	Casting method (5 wt.% H_2_SO_4_)	81.4	2.5	7.8	[38]
	Tunicate nanowhiskers (T-NWs: 15 *v*/*v*%) reinforced ACC films	Casting method (5 wt.% H_2_SO_4_)	137.1 **+68%**	9.8 **+392%**	4.1 **−47%**	
	Cotton nanowhiskers (C-NWs: 15 *v*/*v*%) reinforced ACC films	Casting method (5 wt.% H_2_SO_4_)	127.4 **+56%**	7.2 **+188%**	4.3 **−45%**	
Cotton linter pulps (α-cellulose ≥ 95%) (DP: 617) *	RC film (reference without reinforcement)	Casting method (5 wt.% H_2_SO_4_)	98.4	3.93	8.9	[54]
	Ramie (RA) fibers (delignified, cleaned, cut into short fibers in 5 nm lengths) (RA: 15 wt.%) reinforced ACC films	Casting method (5 wt.% H_2_SO_4_)	124.3 **+26%**	5.25 **+33%**	4.9 **+56%**	
	Ramie (RA) fibers (delignified, cleaned, cut into short fibers in 5 nm lengths) (RA: 25 wt.%) reinforced ACC films	Casting method (5 wt.% H_2_SO_4_)	83.0 **−15%**	5.94 **+51%**	2.5 **−72%**	
Cotton linter pulps (DP: 620)	RC film (reference without reinforcement)	Casting method (5 wt.% H_2_SO_4_)	48.1	4.21	2.2	[56]
	Wet spinning of RC fibers (RCF) in a NaOH–urea solution and cut into ~1 mm lengths: (5 wt.% RCF) reinforced ACC films	Casting method (5 wt.% H_2_SO_4_)	76.0 **+58%**	6.9 **+64%**	2.9 **+32%**	
Cotton linter pulps (DP: 617) *	RC gels (reference without reinforcement)	Injection method (running H_2_O)	0.4	(unspecified)	56	[55]
	Cellulose nanowhiskers (CNW powder: 50 wt. %) reinforced ACC gels	Injection method (running H_2_O)	0.7 **+75%**	(unspecified) **(N/A)**	55 **−1.8%**	
	Cotton fabrics (spun from 20 s Ne Rotor yarn) (plain weave type) (reference without full dissolved cellulose matrix)		216.28 kPa	7.32	18.04	[40]
Viscose fibers (DP: unspecified)	Cotton fabrics (spun from 20 s Ne Rotor yarn) (twill weave type) reinforced ACCs dip-padded with 1.5% of fully dissolved cellulose matrix	Dip-padding method (H_2_O)	219.12 kPa **+1%**	4.22 **−42%**	26.98 **+50%**	
	Cotton fabrics (spun from 20 s Ne Rotor yarn) (twill weave type) (reference without fully dissolved cellulose matrix)		208.40	8.63	16.13	
Viscose fibers (DP: unspecified)	Cotton fabrics (spun from 20 s Ne Rotor yarn) (twill weave type) reinforced ACCs dip-padded with 1.5% of fully dissolved cellulose matrix	Dip-padding method (H_2_O)	210.68 kPa **+0.5%**	4.67 **−46%**	25.55 **+58%**	
	Cotton fabrics (spun from 20 s Ne Rotor yarn) (satin weave type) (reference without fully dissolved cellulose matrix)		203.88 kPa	11.71	13.86	
Viscose fibers (DP: unspecified)	Cotton fabrics (spun from 20 s Ne Rotor yarn) (satin weave type) reinforced ACCs dip-padded with 1.5% of fully dissolved cellulose matrix	Dip-padding method (H_2_O)	208.91 kPa **+3%**	5.40 **−54%**	17.41 **+26%**	
Cotton linter pulp (DP: unspecified)	RC film from 4 wt.% alkali/urea/cellulose (AUC) matrix solution of cotton linter pulp (reference without reinforcement)	Casting method (5 wt.% H_2_SO_4_)	111.0	3.2	12.0	[39]
	TEMPO-oxidized cellulose nanofibrils (TOCN) from never-dried softwood bleached kraft pulp (SBKP) (~90% α-cellulose and ~10% hemicelluloses): (TOCN: 1 wt. %) reinforced ACCs with 4 wt.% AUC matrix solution	Casting method (5 wt.% H_2_SO_4_)	167.0 **+50%**	6.2 **+94%**	10.0 **−17%**	
	Birch (B) fiber sheets (prepared from birch wood pulps by sheet forming) (reference without the impregnation of dissolved cellulose matrices of the D pulps)		9.24	1.05	1.36	This study
Sulfite dissolving (D) pulp (DP: 580.73)	Birch (B) fiber sheets (prepared from birch wood pulps by sheet forming) reinforced ACCs with the impregnation of 1.5 wt.% dissolved cellulose matrix	Vacuum-filtration-assisted impregnation method (deionized H_2_O)	58.4 **+532%**	2.80 **+167%**	7.43 **+446%**	
	Birch (B) fiber sheets (prepared from birch wood pulps by sheet forming) reinforced ACCs with the impregnation of a 2.0 wt.% dissolved cellulose matrices of the D pulps)	Vacuum-filtration-assisted impregnation method (deionized H_2_O)	56.21 **+508%**	3.66 **+248%**	4.62 **+240%**	
	Abaca (A) fiber sheets (prepared from abaca leaf-based pulps by sheet forming) (reference without the impregnation of dissolved cellulose matrices of the D pulps)		32.76	1.68	4.44	
Sulfite dissolving (D) pulp (DP: 580.73)	Abaca fiber sheets (A) (prepared from abaca leaf-based pulps beforehand) reinforced ACCs with the impregnation of a 1.5 wt.% dissolved cellulose matrices of the D pulps)	Vacuum-filtration-assisted impregnation method (deionized H_2_O)	39.68 **+21%**	2.27 **+35%**	4.5 **+1%**	
	Abaca fiber sheets (A) (prepared from abaca leaf-based pulps beforehand) reinforced ACCs with the impregnation of a 2.0 wt.% dissolved cellulose matrices of the D pulps)	Vacuum-filtration-assisted impregnation method (deionized H_2_O)	54.93 **+68%**	1.82 **+8%**	14.11 **+218%**	
	Northern softwood (N) fiber sheets (prepared from the N wood pulps by sheet forming) (reference without the impregnation of the dissolved cellulose matrices of the D pulps)		15.64	1.4	2.59	
Sulfite dissolving (D) pulp (DP: 580.73)	Northern softwood (N) fiber sheets (prepared from the N wood pulps by sheet forming) reinforced ACCs with the impregnation of a 1.5 wt.% dissolved cellulose matrices of the D pulps)	Vacuum-filtration-assisted impregnation method (deionized H_2_O)	35.84 **+129%**	1.93 **+38%**	4.77 **+84%**	
	Northern softwood (N) fiber sheets (prepared from the N wood pulps by sheet forming) reinforced ACCs with the impregnation of a 2.0 wt.% dissolved cellulose matrices of the D pulps)	Vacuum-filtration-assisted impregnation method (deionized H_2_O)	66.43 **+325%**	1.86 **+33%**	14.65 **+466%**	
(*)	The DP values were calculated only for Table 1, based on their reported number-average molecular weight (Mη = 1 × 10^5^) by calculation from the Mη of natural cellulose, which is 162.1406 g/mol.					[63]

**Table 2:** Crystallinity index *Crl* (%) of the birch (B), abaca (A), and northern softwood (N) fiber sheets, fabricated all-cellulose bio-composite samples (B_1.5_, A_1.5_, N_1.5_ and B_2.0_, A_2.0_, N_2.0_), and prepared films (F_1.5_, F_2.0_) from the fully dissolved 1.5 wt.% and 2.0 wt.% cellulose matrix solutions.

Sample	Crystalline Index *Crl* (%)
Birch fiber sheets (B)	73.1
All-cellulose composite (ACC) (B_1.5_)	44.4
All-cellulose composite (ACC) (B_2.0_)	58.3
Abaca fiber sheets (A)	76.8
All-cellulose composite (ACC) (A_1.5_)	60.6
All-cellulose composite (ACC) (A_2.0_)	61.5
Northern softwood fiber sheets (N)	78.3
All-cellulose composite (ACC) (N_1.5_)	59.2
All-cellulose composite (ACC) (N_2.0_)	66.2
Regenerated cellulose film (F_1.5_)	43.7
Regenerated cellulose film (F_2.0_)	43.9

**Table 3:** Structural and mechanical properties of the fabricated fiber sheets (B, A, N) and ACCs.

Samples	Thickness (µm)	Apparent Density (g/cm^3^)	Stress at Break (MPa)	Elastic Modulus (GPa)	Strain at Break (*%*)
B (birch fiber sheet)	138 ± 3	0.65	9.24 ± 3.4	1.05 ± 0.38	1.36 ± 0.43
ACC, B_1.5_	102 ± 1	1.05	58.04 + 2.93	2.80 ± 0.41	7.43 ± 1.57
ACC, B_2.0_	145 ± 5	1.15	56.21 ± 5.5	3.66 ± 0.38	4.62 ± 0.98
A (abaca fiber sheet)	136 ± 4	0.63	32.76 ± 1.74	1.68 ± 0.23	4.44 ± 0.24
ACC, A_1.5_	129 ± 3	0.81	39.68 ± 4.02	2.27 ± 0.42	4.5 ± 1.26
ACC, A_2.0_	142 ± 3	0.97	54.93 ± 5.01	1.82 ± 0.22	14.11 ± 2.5
N (NSW fiber sheet)	131 ± 1	0.62	15.64 ± 0.94	1.4 ± 0.05	2.59 ± 0.18
ACC, N_1.5_	139 ± 7	1.05	35.84 ± 6.76	1.93 ± 0.11	4.77 ± 1.08
ACC, N_2.0_	117 ± 3	1.38	66.43 ± 4.2	1.86 ± 0.15	14.65 ± 1.51

## Data Availability

Data are contained within the article.
